# Basic and Clinical Evidence for Perioperative Immunotherapy in Resectable HNSCC

**DOI:** 10.3390/cancers18111845

**Published:** 2026-06-04

**Authors:** Shota Sakaue, Michihisa Kono, Takumi Kumai, Takahiro Inoue, Hisataka Ominato, Miki Takahara

**Affiliations:** 1 Department of Otolaryngology Head & Neck Surgery, Asahikawa Medical University, Midorigaoka-Higashi 2-1-1-1, Asahikawa 078-8510, Japan; syouta0701@asahikawa-med.ac.jp (S.S.); mkono@asahikawa-med.ac.jp (M.K.); inotaka9242@asahikawa-med.ac.jp (T.I.); h-ominato@asahikawa-med.ac.jp (H.O.); miki@asahikawa-med.ac.jp (M.T.); 2 Department of Innovative Head & Neck Cancer Research and Treatment, Asahikawa Medical University, Asahikawa 078-8510, Japan

**Keywords:** perioperative immunotherapy, head and neck squamous cell carcinoma, immune checkpoint inhibitor, PD-1, KEYNOTE-689, NIVOPOSTOP

## Abstract

**Background**: Perioperative immunotherapy has emerged as a promising strategy for improving outcomes in patients with resectable head and neck squamous cell carcinoma (HNSCC). However, its biological rationale, clinical evidence, and optimal implementation remain incompletely defined. **Methods**: We conducted a narrative review of the current literature, integrating preclinical and clinical evidence on perioperative immune checkpoint inhibitor (ICI) therapy in resectable HNSCC, with particular attention to the immunological impact of surgery, tumor-draining lymph nodes, and treatment sequencing. **Results**: Neoadjuvant immunotherapy exploits the presence of intact tumor antigen and preserved lymphatic architecture, enabling broad T-cell priming, clonal expansion, and systemic immune memory formation. In contrast, adjuvant immunotherapy primarily targets residual microscopic disease and relies on preexisting tumor-reactive T cells, suggesting that these strategies are biologically distinct. Early-phase clinical trials have demonstrated the safety and feasibility of perioperative ICIs, with evidence of pathological responses. Recent phase III trials, including KEYNOTE-689 and NIVOPOSTOP, have provided practice-relevant evidence supporting the integration of ICIs into perioperative treatment, demonstrating improved event-free survival and clinical outcomes in selected populations. Several challenges remain, including optimal patient selection, lack of validated biomarkers, and treatment sequencing after perioperative ICI exposure. **Conclusions**: Perioperative immunotherapy represents a promising practice-relevant strategy in resectable HNSCC. Further studies are required to refine patient selection, develop predictive biomarkers, and optimize treatment sequencing to maximize clinical benefit.

## Simple Summary

Perioperative immunotherapy has emerged as a promising strategy for improving outcomes in resectable head and neck squamous cell carcinoma (HNSCC). This review summarizes the biological rationale and clinical evidence supporting the use of immune checkpoint inhibitors (ICIs) before and after surgery. Neoadjuvant immunotherapy may enhance antitumor immunity by preserving tumor antigens and tumor-draining lymph nodes, thereby promoting T-cell priming, clonal expansion, and systemic immune memory. In contrast, adjuvant immunotherapy primarily targets residual microscopic disease after definitive local treatment. Early-phase studies demonstrated the safety and feasibility of perioperative ICIs, with encouraging pathological responses. Recently, phase III trials such as KEYNOTE-689 and NIVOPOSTOP provided practice-relevant evidence showing improved outcomes with perioperative pembrolizumab or nivolumab-based strategies. However, several challenges remain, including optimal patient selection, biomarker development, and treatment sequencing after perioperative ICI exposure.

## 1. Introduction

Head and neck squamous cell carcinoma (HNSCC) is one of the leading causes of cancer-related morbidity and mortality worldwide and often presents at locoregionally advanced stages [1,2,3,4]. For patients with resectable disease, curative treatment has traditionally focused on surgery, followed by postoperative radiotherapy (RT) or chemoradiotherapy (CRT) according to pathological risk factors, such as positive margins, extranodal extension, nodal burden, perineural invasion, lymphovascular invasion, and depth of invasion [5,6,7,8]. This multimodal, surgery-based approach remains the standard treatment for resectable locally advanced HNSCC.

Despite advances in surgical techniques, reconstruction, perioperative management, and adjuvant therapies, recurrence remains a significant clinical concern in resected HNSCC. Even after complete resection and appropriate postoperative RT or CRT, a significant number of patients experience locoregional recurrence or distant metastases. This is especially true for patients with high-risk disease, for whom the current standard treatment often fails to achieve long-term control [6,7,9,10,11]. Therefore, effective strategies to improve the prognosis of patients with resectable disease are warranted.

Over the past decade, immune checkpoint inhibitors (ICIs), particularly those targeting the PD-1/PD-L1 axis, have transformed the therapeutic landscape for patients with recurrent or metastatic HNSCC [5,12,13,14,15,16]. These findings indicate that antitumor immunity is effective in HNSCC, raising expectations that immunotherapies may also improve outcomes in resectable HNSCC. Indeed, perioperative administration of PD-1 inhibitors has been shown to improve outcomes in other malignancies, such as renal cell carcinoma and lung cancer [17,18,19,20,21]. In HNSCC, the phase III KEYNOTE-689 trial showed that adding neoadjuvant and adjuvant pembrolizumab to the standard of care improved event-free survival in patients with resectable locally advanced HNSCC [22]. Similarly, the phase III NIVOPOSTOP trial revealed that adding nivolumab to postoperative cisplatin radiotherapy improved outcomes in patients with pathologically high-risk resected disease [23]. These studies suggest that immunotherapy is effective in both recurrent or metastatic HNSCC and resectable disease with curative intent. Conversely, the addition of ICIs to chemoradiotherapy has not demonstrated clinical benefit, as evidenced by the negative phase III JAVELIN Head and Neck 100 and KEYNOTE-412 trials in the definitive setting [24,25]. In this review, we provide an overview of the basic and clinical evidence for perioperative immunotherapy in HNSCC, as well as future perspectives, with consideration of the impact of surgery on the immune system.

## 2. Limitations of Surgery-Based Treatment: Residual Risk and Perioperative Immune Suppression

In most cases, surgery is a central component of treatment to achieve locoregional control in resectable HNSCC. However, even after complete resection, the presence of major risk factors, such as extranodal extension, is associated with an increased risk of locoregional or distant recurrence; therefore, postoperative radiotherapy or chemoradiotherapy is typically indicated. Although these adjuvant therapies improve disease-free survival, recurrence still occurs in a proportion of patients [6,7,9,10,11], indicating that complete macroscopic resection does not necessarily mean complete eradication of disease.

A major limitation of surgery-based treatment is the persistence of residual microscopic disease. Microscopic tumors may remain in the operative bed, regional lymph nodes, or distant sites, and these foci may not be eliminated by local therapy. Postoperative RT or CRT could reduce this risk of recurrence; however, patients with adverse pathological features continue to experience substantial rates of locoregional relapse and distant metastasis [6,7,9,10,11]. Although distant micrometastases are difficult to predict, pathological evaluation of lymph nodes has identified micrometastases in approximately 10% of cases initially classified as N0 [26]. Therefore, even with optimal surgical resection, recurrence due to micrometastases or microscopic residual disease cannot be completely prevented.

The perioperative period is recognized as an immunologically significant phase. Surgical stress can induce transient but clinically significant immunosuppression via inflammatory, neuroendocrine, and cellular mechanisms, which may hinder effective antitumor immunity [27,28,29]. Although B cells are relatively unaffected, the number and function of cytotoxic and helper T cells decrease, while regulatory T cells increase. Endogenous corticosteroids released in response to surgical stress, as part of the host recovery process, contribute to these changes. While this immunomodulation is a physiological response necessary to prevent excessive inflammation and promote wound healing at the surgical site, it may have detrimental effects on antitumor immunity. On the other hand, arginine is an amino acid required for T-cell proliferation, and perioperative arginine supplementation has been reported to improve disease-specific survival [30]. Therefore, enhancing antitumor immunity during the perioperative period may be beneficial for improving long-term outcomes in HNSCC.

Cervical lymphatics and tumor-draining lymph nodes serve not only as sites of tumor spread requiring resection but also as key organs for T-cell priming and activation [31,32,33,34]. Definitive surgery, especially when combined with nodal dissection, can affect both the tumor burden and the local immune network that supports antitumor immune priming and maintenance [35,36]. If effective immune priming depends on preserved tumor antigen exposure within the tumor-draining lymphatic system, immunotherapy may be most effective when administered prior to surgery. In the following section, we review the basic evidence supporting perioperative immunotherapy.

## 3. Biological Rationale for Perioperative Immunotherapy

### 3.1. Conceptual Basis for Neoadjuvant Immunotherapy

**Figure 1:**
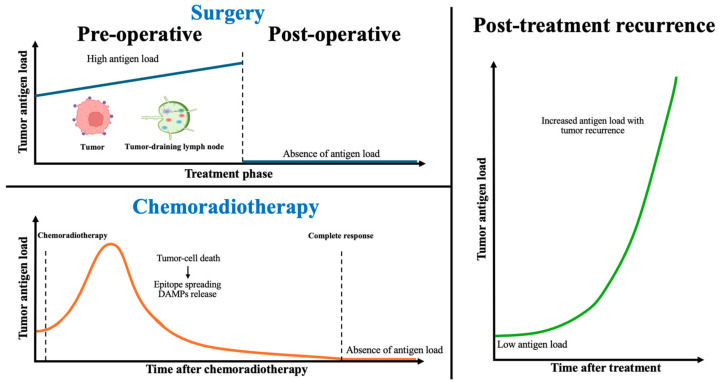
Conceptual changes in tumor antigen load and antigen presentation during perioperative treatment in HNSCC.

From the perspective of tumor antigen load, abundant tumor-derived antigens are present prior to treatment, whereas surgical resection markedly reduces the availability of tumor antigens. During chemoradiotherapy, tumor cell destruction transiently releases tumor antigens together with damage-associated molecular patterns (DAMPs), which may function as endogenous adjuvants and facilitate epitope spreading. However, as tumor burden decreases, the antigen load gradually declines over time. In contrast, tumor recurrence is associated with reaccumulation of tumor antigens in proportion to tumor volume (Figure 1).

Perioperative immunotherapy is immunologically attractive because it exploits a treatment window in which the tumor, the tumor microenvironment including immune cells, and the tumor-draining lymphatic network are still preserved. In the neoadjuvant setting, immune checkpoint blockade is delivered while abundant tumor antigens remain present, allowing ongoing antigen release, uptake by antigen-presenting cells, and priming of tumor-reactive T cells [37,38,39,40,41,42]. This differs fundamentally from a purely adjuvant strategy, in which most tumor and its antigen has already been removed and the opportunity for broad de novo priming may be reduced [42,43].

- The conceptual basis for perioperative immunotherapy is consistent with the cancer-immunity cycle. Effective antitumor immunity requires release of cancer antigens, presentation by dendritic cells, priming and activation of T cells, trafficking of effector cells, and recognition and killing of cancer cells [37,38]. Neoadjuvant immunotherapy preserves the early steps of this process because the intact tumor continues to provide antigenic stimulation. By contrast, postoperative treatment may depend more heavily on pre-existing immunity and less on the generation of new tumor-specific T-cell responses [42,43].
- A particularly important preclinical study by Friedman et al. showed that neoadjuvant PD-1 blockade broadened tumor antigen-specific T-cell responses, including subdominant clones, and generated immune memory capable of protecting against tumors lacking the previously dominant antigen [44]. Because resistance to immunotherapy may arise through antigen loss or narrowing of the immune repertoire, the induction of subdominant T-cell clones in the presence of a large amount of tumor antigen during PD-1 blockade may enhance immune surveillance. Thus, neoadjuvant therapy is not simply “earlier immunotherapy”, but it may qualitatively reshape the antitumor T-cell response. Recent reviews further support the concept that neoadjuvant immunotherapy may enhance systemic antitumor immunity and durable immune memory [44,45].

### 3.2. Antigen Availability and Cross-Presentation

The effectiveness of ICIs depends on the presence of recognizable tumor antigens and the capacity of antigen-presenting cells to initiate productive T-cell responses [39,40,41,42]. Foundational work in tumor immunology established that neoantigens derived from tumor-specific mutations can serve as critical targets for immune recognition and for the efficacy of ICIs [39,40,41,42]. A second essential requirement is antigen cross-presentation by dendritic cells, especially the cDC1/Batf3-dependent lineage [46,47,48,49]. This dendritic cell subset is central to cross-priming CD8+ T cells against tumor antigens. Multiple studies have shown that effective antitumor T-cell priming and responsiveness to ICIs depend on intact cDC1 function and crosstalk between T cells and dendritic cells within the tumor microenvironment, particularly through IFN-γ and IL-12 signaling [46,47,48,49,50]. Earlier work also demonstrated that BATF3-dependent dendritic cells are indispensable for the efficacy of anti-PD-1-based immunotherapy by enabling effective cross-priming of tumor-specific CD8+ T cells [51]. In the neoadjuvant setting, the tumor remains in place as a source of antigens, while the draining lymphatic network and antigen-presenting machinery including dendritic cells remain intact. This favors broad priming of tumor-specific T cells before the local immune architecture is disrupted by definitive treatment [43,44,52]. These observations strongly support the biological rationale for neoadjuvant immunotherapy.

### 3.3. Central Role of Tumor-Draining Lymph Nodes in Antitumor Immunity

Recent preclinical and translational studies suggest that tumor-draining lymph nodes are not merely passive bystanders, but may serve as important immunological sites involved in responses to checkpoint blockade [33,34,35]. These nodes serve as hubs where tumor antigens are presented by antigen-presenting cells to naïve or stem-like T cells, allowing clonal expansion and differentiation into effector populations. A key study by Huang et al. demonstrated that a subset of tumor-specific memory-like CD8+ T cells in draining lymph nodes behaves as a bona fide responder population to PD-1/PD-L1 blockade [34]. This finding supports the hypothesis that checkpoint inhibition may involve not only reinvigoration of exhausted intratumoral T cells, but also activation of tumor-reactive T-cell populations residing within the draining nodal compartment. These observations support a model in which meaningful immune reinvigoration occurs both within the tumor microenvironment and in tumor-draining lymph nodes, suggesting that preservation of tumor-draining lymph node function before extensive surgery or nodal irradiation may contribute to the efficacy of checkpoint blockade, although direct causal evidence in human HNSCC remains limited [33,34,35].

Saddawi-Konefka et al. provided direct evidence in an experimental HNSCC model that cervical lymphatic-preserving treatment sequencing can unleash cDC1-dependent antitumor immunity [35]. These findings support the concept that preserved lymphatic architecture may influence perioperative immunotherapy responses, and that treatment order in HNSCC is not merely logistical but immunologically meaningful. Preclinical models showed that oral but not subcutaneously injected tumors generated stronger lymph-node T-cell responses and better anti-PD-1 efficacy, supporting the idea that the locoregional lymph-node immunity axis in HNSCC is important to elicit antitumor immunity [31]. This interpretation is consistent with HNSCC-focused translational reviews that emphasize paired lymph node analyses rather than tumor analysis alone [53,54]. These findings suggest that preservation of the tumor-draining lymph node axis including BATF3-dependent dendritic cells may contribute to perioperative immunotherapy responsiveness [49,50,51]. Several lines of human evidence are beginning to support the clinical relevance of tumor-draining lymph nodes in HNSCC. Activation of tumor-reactive T cells within draining lymph nodes has been associated with longer overall survival, suggesting that immune activation in the nodal compartment may contribute to antitumor control [55]. In addition, prior neck dissection has been reported to attenuate the efficacy of ICIs at recurrence or metastasis, indirectly supporting the importance of preserved cervical lymphatic and nodal immune function [56]. As perioperative immunotherapy becomes more widely implemented, analyses of cervical lymph nodes obtained after neoadjuvant ICI therapy may provide an important opportunity to clarify the immunological and clinical significance of tumor-draining lymph nodes in human HNSCC.

### 3.4. Systemic Immunity, Clonal Remodeling, and Immune Memory

**Figure 2:**
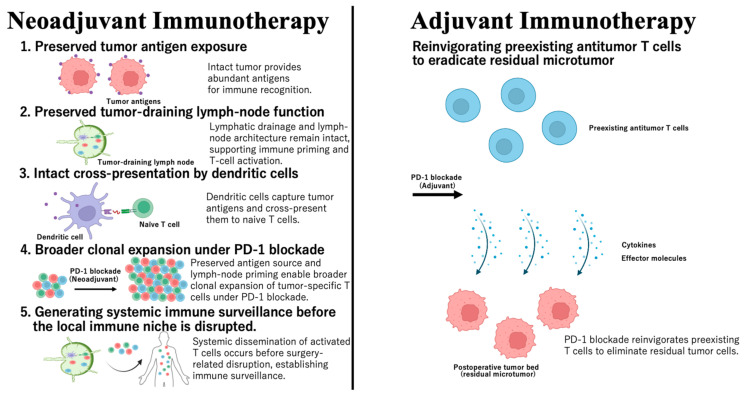
Biological rationale for neoadjuvant and adjuvant immunotherapy in resectable HNSCC.

Peripheral and intratumoral T-cell studies have shown that checkpoint blockade can remodel the clonotypic composition of responding T cells, including expansion or replacement by novel tumor-reactive clonotypes [57]. This supports the concept that successful immunotherapy may recruit or amplify a broader systemic antitumor repertoire rather than merely transiently reinvigorating preexisting tumor-infiltrating lymphocytes. Because relapse after curative-intent local treatment is often driven by occult residual disease rather than uncontrolled primary tumor, surveillance by tumor-reactive T cells against residual microscopic disease induced by ICIs after surgery may be warranted [44,52,57]. Collectively, these findings support the biological rationale for neoadjuvant immunotherapy in HNSCC, particularly in the context of preserved antigen exposure and tumor-draining lymph-node function. In contrast, adjuvant immunotherapy primarily targets residual microscopic disease after definitive local therapy (Figure 2).

## 4. Clinical Evidence for Perioperative Immunotherapy

### 4.1. Early Proof-of-Concept Studies

Clinical development of perioperative immunotherapy in resectable HNSCC began with relatively small early-phase neoadjuvant PD-1 blockade studies designed primarily to evaluate safety, feasibility, and preliminary biological activity [36,58,59,60]. These studies consistently showed that preoperative immunotherapy could be administered without compromising surgery, while also demonstrating measurable pathological treatment effect after only short exposure. A landmark phase II study by Uppaluri et al. evaluated neoadjuvant followed by adjuvant pembrolizumab in resectable HPV-unrelated HNSCC and showed pathological tumor response after a single neoadjuvant dose [36]. Wise-Draper et al. extended this approach in a larger phase II trial, reporting encouraging 1-year disease-free survival outcomes relative to historical controls with neoadjuvant and adjuvant pembrolizumab. Pathological response correlated with improved DFS, however, interpretation is limited by the non-randomized design and modest sample size [58]. In addition, major pathological response and pathological complete response should currently be regarded as exploratory translational endpoints that may correlate with favorable clinical outcomes, although further validation in larger prospective studies with long-term follow-up is required.

Parallel nivolumab-based studies produced similar proof-of-concept signals. In CheckMate 358, neoadjuvant nivolumab was generally safe and induced pathological regressions in both HPV-positive and HPV-negative disease [59]. The IMCISION trial then showed that nivolumab plus ipilimumab could induce major pathological responses in a subset of patients, further supporting the feasibility and biological activity of intensified perioperative immune priming, although patient numbers were limited [60].

### 4.2. Surgical Safety and Feasibility

A concern has been whether neoadjuvant immunotherapy delays surgery, worsens wound healing, or increases perioperative morbidity. Across early trials and pooled analyses, the overall safety signal has been reassuring [61,62]. A systematic review and meta-analysis found low rates of severe preoperative treatment-related adverse events and no clear signal of systematic surgical delay attributable to neoadjuvant PD-1/PD-L1 blockade in resectable HNSCC [62]. In oral cavity surgery, neoadjuvant pembrolizumab did not increase surgical complications attributable to preoperative treatment [61], supporting the practical integration of a short neoadjuvant ICI without undermining resectability, reconstruction planning, or postoperative therapy.

Neoadjuvant ICI therapy combined with surgery offers a unique opportunity for direct biological and pathological assessment of immunotherapy using paired pre- and post-treatment tissue samples in HNSCC. This approach enables detailed analyses of immune cell infiltration, gene expression changes, and treatment-induced remodeling of the tumor microenvironment [58]. Pathological complete response and major pathological response have become central translational endpoints in perioperative immunotherapy studies [58,63,64].

### 4.3. Intensified Perioperative Approaches

Although neoadjuvant PD-1 blockade induces antitumor immunity, pathological response rates were still modest; therefore, various strategies to further augment antitumor immunity have been investigated. To further improve oncologic outcomes, dual checkpoint blockade has been tested. IMCISION and related studies suggest that combining nivolumab with ipilimumab, or durvalumab with tremelimumab, may produce deeper pathological responses in selected patients, although toxicity and complexity increase accordingly [60,65]. Radiotherapy can promote antigen release, increase MHC expression, and induce immunogenic cell death, thereby potentially enhancing the efficacy of checkpoint blockade [66,67]. In a phase Ib study, neoadjuvant immunoradiotherapy resulted in a high rate of pathological downstaging and pathological complete response without delaying surgery [68]. This is one of the strongest clinical demonstrations that local radiation can potentiate perioperative immune responses in HNSCC [66,67,68]. Chemotherapy may likewise increase tumor antigen availability and alter suppressive immune populations, although its effects depend strongly on the regimen and schedule [69,70]. Recent prospective and retrospective studies suggest that neoadjuvant chemoimmunotherapy can achieve higher pathological complete response and major pathological response rates than monotherapy, although toxicity may be higher in some series [69,70,71,72,73,74,75]. Nevertheless, systematic reviews support that chemoimmunotherapy may represent a promising neoadjuvant approach, although the currently available evidence is largely derived from early-phase studies with relatively limited follow-up [72,74,75,76].

### 4.4. Practice-Relevant Phase III Evidence

The major turning point in the field was KEYNOTE-689. In this phase III trial, the addition of neoadjuvant and adjuvant pembrolizumab to standard care significantly improved event-free survival in a broader population of resectable locally advanced HNSCC, without compromising the surgical completion [22]. This was the first definitive phase III evidence showing that a perioperative immunotherapy improves clinically meaningful outcomes in resectable HNSCC. A second landmark study, NIVOPOSTOP, evaluated a different concept, in which nivolumab was added to postoperative cisplatin-based radiotherapy to eradicate residual microscopic disease after definitive surgery. This trial showed that adding ICI to postoperative chemoradiotherapy improved outcomes in high-risk resected locally advanced HNSCC, providing evidence that immunotherapy can also improve outcomes when integrated into postoperative treatment for selected high-risk patients [23].

Comparative reviews have emphasized that KEYNOTE-689 (neoadjuvant and adjuvant approaches) and NIVOPOSTOP (adjuvant approach) should not be regarded as interchangeable studies or interpreted as providing direct comparative evidence [77]. Instead, they likely define two related but distinct models: perioperative immune priming for a broader resectable population, and postoperative immune intensification for a pathologically high-risk subgroup [77]. Whereas neoadjuvant immunotherapy aims to prime systemic antitumor immunity in the presence of intact tumor antigen and preserved tumor-draining lymphatic architecture, postoperative immunotherapy primarily targets residual microscopic disease after definitive local therapy [77,78]. This distinction is clinically important and should be acknowledged when interpreting the current evidence. It also remains unclear whether perioperative or adjuvant ICI therapy provides additional benefit in low-risk HNSCC, and whether perioperative ICI is truly effective in high-risk disease. In addition, whether neoadjuvant ICIs alone is sufficient warrants further investigation.

Although the addition of ICIs to chemotherapy may enhance tumor antigen release and contribute to improved pathological responses, it may also increase the risk of adverse events such as pneumonitis [22] and renal dysfunction [23], which can lead to treatment discontinuation. Thyroid dysfunction was among the most common immune-related adverse events associated with ICIs and was generally manageable. In contrast, renal dysfunction may be induced by both ICIs and cisplatin, and this toxicity is clinically important because it may compromise the feasibility of subsequent systemic chemotherapy. Although the overall safety profiles of the NIVOPOSTOP and KEYNOTE-689 trials were considered acceptable, these safety considerations warrant further emphasis.

### 4.5. Adjuvant Immunotherapy and Its Clinical Implications

Before NIVOPOSTOP, postoperative immunotherapy had not demonstrated convincing efficacy in HNSCC, and much of the available evidence was limited to early-phase or non-randomized studies [78,79]. In the randomized IMvoke010 study, maintenance atezolizumab after multimodal definitive treatment for high-risk locally advanced HNSCC did not improve clinical outcomes versus placebo, indicating that postoperative PD-L1 blockade could not provide benefit in an unselected population [78]. Because the addition of ICIs to chemoradiotherapy failed to demonstrate a significant survival benefit in the phase III JAVELIN Head and Neck 100 and KEYNOTE-412 trials [24,25], the incremental benefit of adding ICIs to standard curative-intent treatment has been difficult to establish. In this context, the positive results of the NIVOPOSTOP trial represent a landmark finding in the field of curative-intent treatment for HNSCC. By demonstrating that nivolumab added to postoperative cisplatin-based radiotherapy improved outcomes in high-risk, resected locally advanced HNSCC, this study moved adjuvant immunotherapy from an area of uncertainty to one supported by robust clinical evidence [23,77]. Importantly, this suggests that postoperative immune intensification may be effective when applied to a pathologically selected high-risk population with microscopic residual disease, rather than broadly across all patients after definitive treatment.

Because the control arm of the NIVOPOSTOP trial consisted of postoperative chemoradiotherapy alone, it remains unclear whether ICIs alone would provide sufficient therapeutic benefit in postoperative high-risk patients. The IMvoke010 study included approximately 30% of patients who received ICIs as maintenance therapy following surgical treatment [78]. Although subgroup analyses limited to surgically treated patients have not been reported, the lack of an observed survival benefit suggests that postoperative ICI monotherapy may be insufficient in this setting. In contrast, the positive findings of the NIVOPOSTOP trial support the possibility that concurrent integration of ICIs with postoperative chemoradiotherapy, rather than postoperative ICI maintenance alone, is required to achieve meaningful therapeutic benefit only in high-risk HNSCC. In addition, when interpreted together with the negative results of the JAVELIN Head and Neck 100 trial [24], which similarly evaluated the addition of ICIs to chemoradiotherapy in high-risk disease, these findings suggest that ICIs may be more effective in the setting of minimal residual disease following surgical tumor debulking, particularly when combined with postoperative chemoradiotherapy. Consistent with this concept, clinical studies in melanoma have suggested that a high tumor burden may reduce the efficacy of immunotherapy [80].

## 5. Conclusions

**Figure 3:**
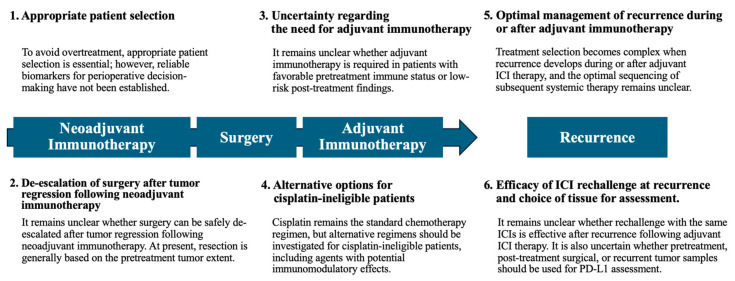
Remaining challenges and future perspectives for perioperative immunotherapy in resectable HNSCC.

The available basic and clinical evidence, including KEYNOTE-689 and NIVOPOSTOP, supports the efficacy of immunotherapy in resectable HNSCC; however, several issues remain to be addressed (Figure 3). In KEYNOTE-689, all patients underwent postoperative radiotherapy; however, in real-world practice, some patients may have been low risk and potentially curable with surgery alone. Because pathological high-risk features cannot be identified before surgery, imaging-based risk stratification prior to treatment initiation may represent a useful approach for selecting candidates for neoadjuvant ICI therapy. PD-L1 expression, tumor mutational burden, immune signatures, and pathological response each provide valuable information; however, none alone is sufficient for perioperative decision-making to avoid overtreatment [53,54,63,64]. In KEYNOTE-689 and NIVOPOSTOP trials, event-free survival was improved irrespective of PD-L1 expression. Although the overall survival analysis was exploratory and not formally powered for statistical significance, a survival benefit appeared to be more pronounced in patients with CPS ≥ 10 in KEYNOTE-689 [22]. On the other hand, only a small proportion of patients with CPS < 1 or unevaluable PD-L1 expression were included, and therefore the clinical significance of neoadjuvant ICI therapy in PD-L1–low HNSCC remains unclear. In the negative phase III KEYNOTE-412 and JAVELIN Head and Neck 100 trials [24,25], a trend toward greater benefit from ICIs was observed in patients with higher PD-L1 expression; however, a clear survival advantage was not demonstrated. Importantly, different antibody clones were used to assess PD-L1 expression across these studies. We have previously reported that PD-L1 expression levels can vary substantially depending on the antibody clone used for evaluation [81]. Therefore, even when studies appear to assess the same biomarker, caution is warranted when interpreting PD-L1–based analyses across trials employing different antibody clones. At present, the clinical utility of PD-L1 expression as a predictive biomarker for perioperative ICI therapy in HNSCC remains uncertain.

Current or former smokers accounted for approximately 80% of patients in both the NIVOPOSTOP and KEYNOTE-689 trials, and therefore the relationship between smoking history and the efficacy of perioperative immune checkpoint inhibitor therapy remains unclear. Interestingly, hypopharyngeal cancer tended to show relatively poorer outcomes in the KEYNOTE-689 trial, whereas comparatively favorable outcomes were observed in the NIVOPOSTOP trial. In contrast, oral cavity and oropharyngeal cancers appeared to demonstrate more favorable outcomes in KEYNOTE-689. Because the number of patients within each primary tumor site subgroup was limited, further investigation is required to determine whether the efficacy of perioperative immune checkpoint inhibitor therapy differs according to tumor location.

Because HPV-positive (p16-positive) HNSCC accounted for less than 5% of cases in both the NIVOPOSTOP and KEYNOTE-689 trials [22,23], the clinical significance and efficacy of perioperative immunotherapy in HPV-positive HNSCC remain unclear. In the recurrent or metastatic setting, HPV status has not been shown to significantly influence the efficacy of ICIs [82]. Therefore, treatment strategies for perioperative ICIs cannot be modified on the basis of HPV status alone. Although HPV-derived antigens are considered highly immunogenic because they originate from viral proteins, HPV-negative HNSCCs also harbor a relatively high neoantigen burden, partly due to frequent TP53 mutations, and are generally classified as immunologically “hot” tumors. This may explain why the immunological advantage of HPV-positive disease has not translated into a clear difference in ICI efficacy.

It remains to be determined whether perioperative immunotherapy is necessary in patients with high levels of tumor-infiltrating lymphocytes in pretreatment samples or whether adjuvant therapy, including immunotherapy, is required in patients with low-risk pathological features or evidence of antitumor immune activation in post-treatment specimens. At present, the patient populations most suitable or unsuitable for neoadjuvant ICI therapy in stage III or higher HNSCC remain poorly defined. Nevertheless, neoadjuvant ICIs should likely not be applied to early-stage disease that can already be effectively managed with established standard treatments, including surgery. In cases of rapidly progressive disease, neoadjuvant cytotoxic chemotherapy may be preferentially selected over ICI monotherapy because immediate tumor reduction may be clinically necessary to prevent deterioration in airway patency, swallowing function, or general condition. However, robust evidence supporting optimal treatment selection in such scenarios remains lacking. Future prospective studies are therefore warranted to determine whether clinical parameters, including tumor growth kinetics, symptom progression, functional compromise, and the risk of treatment delay, can be used to guide individualized selection among neoadjuvant ICI monotherapy, combination immunotherapy, and conventional cytotoxic chemotherapy.

With the transition of perioperative ICIs into real-world practice, an important clinical question is whether de-escalated surgery is acceptable for tumors that have regressed after neoadjuvant ICIs. Cao et al. have shown that flap reconstruction could be exempted in most cases with neoadjuvant ICIs with platinum-based doublet chemotherapy [83]. Currently, due to the lack of sufficient evidence regarding de-escalated surgery after neoadjuvant therapy, including ICIs and chemotherapy, surgical resection should generally be planned based on the pretreatment tumor extent defined by imaging. Future strategies may incorporate response-adapted imaging reassessment and the potential for de-escalated surgery according to the degree of tumor regression. Although the critical role of cervical lymph nodes in antitumor immunity has been discussed above, it should be noted that neck dissection of metastatic lymph nodes remains necessary at present, even in the era of adjuvant ICI therapy. We speculate that tumor antigen–mediated T-cell priming within cervical lymph nodes is largely completed within approximately 1–2 weeks after ICI administration [35], after which memory T cells may become systemically distributed. From this perspective, standard neck dissection following neoadjuvant ICI treatment may be acceptable when sufficient time has elapsed after ICI administration. Although super-selective neck dissection targeting only radiologically or pathologically confirmed metastatic lymph nodes may represent a preferable strategy for preserving regional lymphatic immune function, improved imaging modalities for detecting metastatic lymph nodes are required to safely implement such approaches in clinical practice. At present, treatment sequencing strategies designed to preserve and exploit lymph node immune function, such as neoadjuvant approaches, are likely to represent the most clinically applicable strategy.

Finally, the optimal management of patients who experience recurrence during or after adjuvant ICI therapy remains a challenging clinical issue. At present, the highest level of evidence for recurrent/metastatic HNSCC supports pembrolizumab plus platinum and fluorouracil (FP) or nivolumab for patients with disease progression within 6 months after cisplatin-based therapy [14,15]. However, treatment selection becomes complex when patients have already received pembrolizumab or nivolumab in the perioperative setting. In patients receiving maintenance pembrolizumab monotherapy, options include the addition of FP, switching to cetuximab-based chemotherapy, or, in cases of recent cisplatin exposure, transitioning to nivolumab. Conversely, for patients treated with nivolumab, subsequent options—such as pembrolizumab monotherapy or pembrolizumab plus FP based on CPS, or cetuximab-based chemotherapy—require careful consideration. In patients who have already completed adjuvant ICI therapy, whether to rechallenge with the same ICIs remains a challenging clinical question. Several small studies have suggested that cetuximab-based chemotherapy may retain efficacy following ICI therapy [84]. However, whether its therapeutic efficacy differs according to prior ICI exposure remains unclear, and validation in larger prospective studies is warranted. Even when PD-L1 status is considered as a guide for treatment selection, its expression may differ between primary and recurrent tumors [14]; therefore, it remains unclear which tissue—pretreatment, post-treatment, or recurrent samples—should be used for biomarker assessment. In addition, PD-L1 expression is characterized not only by temporal changes during disease progression and treatment but also by substantial intratumoral heterogeneity within the same tumor specimen [85], suggesting that its utility as a standalone biomarker for perioperative ICI selection remains uncertain.

Although available tissue samples may not necessarily represent the entire tumor because of the marked spatial and temporal inter- and intratumoral heterogeneity of HNSCC, single-cell analyses of recurrent tumor tissues may improve the prediction of ICI responsiveness by more comprehensively characterizing the tumor immune microenvironment, as has been explored in other cancer types [86,87]. However, although single-cell RNA sequencing and spatial transcriptomic analyses may provide highly detailed characterization of the tumor immune microenvironment, their widespread implementation in routine clinical practice remains challenging because of financial and technical limitations. Future studies should focus on treatment stratification using biomarkers that are already clinically applicable, including PD-L1 immunohistochemistry, histopathological evaluation of tumor-infiltrating lymphocytes and tertiary lymphoid structures, and peripheral blood–based immune markers such as the neutrophil-to-lymphocyte ratio.

Overall, the available evidence supports perioperative immunotherapy as a promising and increasingly practice-relevant strategy in resectable HNSCC, although optimal patient selection, biomarker development, and treatment sequencing remain to be defined and warrant further investigation.

## Data Availability

No new data were created or analyzed in this study.

## Abbreviations

The following abbreviations are used in this manuscript:

APC	Antigen-presenting cell
BATF3	Basic leucine zipper ATF-like transcription factor 3
cDC1	Conventional dendritic cell type 1
CCRT	Concurrent chemoradiotherapy
CI	Confidence interval
CPS	Combined positive score
CRT	Chemoradiotherapy
CTLA-4	Cytotoxic T-lymphocyte-associated protein 4
DFS	Disease-free survival
EFS	Event-free survival
FP	Fluorouracil plus platinum
HNSCC	Head and neck squamous cell carcinoma
HPV	Human papillomavirus
ICI	Immune checkpoint inhibitor
IFN-γ	Interferon gamma
IL-12	Interleukin-12
MHC	Major histocompatibility complex
OS	Overall survival
PD-1	Programmed cell death protein 1
PD-L1	Programmed death-ligand 1
RT	Radiotherapy
T-cell	T lymphocyte
TMB	Tumor mutational burden
Treg	Regulatory T cell
